# A multilevel health system intervention for virological suppression in adolescents and young adults living with HIV in rural Kenya and Uganda (SEARCH-Youth): a cluster randomised trial

**DOI:** 10.1016/S2352-3018(23)00118-2

**Published:** 2023-08

**Authors:** Theodore Ruel, Florence Mwangwa, Laura B Balzer, James Ayieko, Marilyn Nyabuti, Wafula Erick Mugoma, Jane Kabami, Brian Kamugisha, Douglas Black, Bridget Nzarubara, Fred Opel, John Schrom, George Agengo, Janet Nakigudde, Hellen N Atuhaire, Josh Schwab, James Peng, Carol Camlin, Starley B Shade, Elizabeth Bukusi, Bill G Kapogiannis, Edwin Charlebois, Moses R Kamya, Diane Havlir

**Affiliations:** Department of Pediatrics (Prof T Ruel MD), Division of HIV, Infectious Diseases and Global Medicine, Department of Medicine (D Black BA, J Schrom MPH, Prof D Havlir MD), Department of Epidemiology and Biostatistics (Prof E Charlebois PhD, S B Shade PhD), and Department of Obstetrics, Gynecology & Reproductive Sciences (Prof C Camlin PhD), University of California, San Francisco, San Francisco, CA, USA; Infectious Diseases Research Collaboration, Kampala, Uganda (F Mwangwa MSc, B Nzarubara MSc, B Kamugisha MSc, J Kabami MPH, H N Atuhaire BMLS); Division of Biostatistics, School of Public Health, University of California, Berkeley, CA, USA (L B Balzer PhD, J Schwab MSc); Kenya Medical Research Institute, Kisumu, Kenya (J Ayieko PhD, M Nyabuti MBchB, W E Mugoma MSc, F Opel BS, G Agengo BSc, Prof E Bukusi PhD); Department of Psychiatry (J Nakigudde PhD) and Department of Medicine (Prof M R Kamya PhD), Makerere University College of Health Sciences, Kampala, Uganda; Department of Biostatistics, University of Washington, Seattle, WA, USA (J Peng MS); Eunice Kennedy Shriver National Institute of Child Health and Human Development, Bethesda, MD, USA (B G Kapogiannis MD)

## Abstract

**Background:**

Social and cognitive developmental events can disrupt care and medication adherence among adolescents and young adults living with HIV in sub-Saharan Africa. We hypothesised that a dynamic multilevel health system intervention helping adolescents and young adults and their providers navigate life-stage related events would increase virological suppression compared with standard care.

**Methods:**

We did a cluster randomised, open-label trial of young individuals aged 15–24 years with HIV and receiving care in eligible clinics (operated by the government and with ≥25 young people receiving care) in rural Kenya and Uganda. After clinic randomisation stratified by region, patient population, and previous participation in the SEARCH trial, participants in intervention clinics received life-stage-based assessment at routine visits, flexible clinic access, and rapid viral load feedback. Providers had a secure mobile platform for interprovider consultation. The control clinics followed standard practice. The primary, prespecified endpoint was virological suppression (HIV RNA <400 copies per mL) at 2 years of follow-up among participants who enrolled before Dec 1, 2019, and received care at the study clinics. This trial is registered with ClinicalTrials.gov, NCT03848728, and is closed to recruitment.

**Findings:**

28 clinics were enrolled and randomly assigned (14 control, 14 intervention) in January, 2019. Between March 14, 2019, and Nov 26, 2020, we recruited 1988 participants at the clinics, of whom 1549 were included in the analysis (785 at intervention clinics and 764 at control clinics). The median participant age was 21 years (IQR 19–23) and 1248 (80·6%) of 1549 participants were female. The mean proportion of participants with virological suppression at 2 years was 88% (95% CI 85–92) for participants in intervention clinics and 80% (77–84) for participants in control clinics, equivalent to a 10% beneficial effect of the intervention (risk ratio [RR] 1·10, 95% CI 1·03–1·16; p=0·0019). The intervention resulted in increased virological suppression within all subgroups of sex, age, and care status at baseline, with greatest improvement among those re-engaging in care (RR 1·60, 95% CI 1·00–2·55; p=0·025).

**Interpretation:**

Routine and systematic life-stage-based assessment, prompt adherence support with rapid viral load testing, and patient-centred, flexible clinic access could help bring adolescents and young adults living with HIV closer towards a goal of universal virological suppression.

**Funding:**

Eunice Kennedy Shriver National Institute of Child Health and Human Development, US National Institutes of Health.

## Introduction

Adolescents and young adults bear a large burden of the HIV epidemic. In 2020, 1·75 million adolescents (aged 10–19 years) were estimated to be living with HIV globally.^1^ As they age, adolescents and young adults with HIV in sub-Saharan Africa face highly dynamic pressures that lead to disruptions in care and poor adherence to treatment, resulting in lower rates of suppression compared with older adults.^2^ As described in the conceptual framework of Sawyer and colleagues^3^ and as recognised by UNAIDS,^4^ cognitive and social role changes interact with social determinants to impact the health and behaviours of adolescents and young adults living with HIV. We developed the SEARCH-Youth intervention using the empirically validated PRECEDE model to identify and accommodate the dynamic challenges faced by adolescents and young adults living with HIV.^5^ The PRECEDE model is based on the idea that health promotion strategies are most effective when they are created with the people affected and when they address predisposing factors, including knowledge, attitudes, or beliefs that affect behaviour; enabling factors, which facilitate change by making the behaviour easier; and reinforcing factors, which include anticipated consequences after a behaviour. Engaging with young people and clinic providers, we developed the SEARCH-Youth intervention, with feature components at patient, provider, and clinic levels. First, a life-stage-based assessment tool aims to change the nature of the clinical interaction and help providers and young people identify facilitators and barriers to care and adherence. Second, young people are offered alternative access options to address barriers to coming to the clinic, including having visits by phone, after hours, or offsite. Third, viral load testing is done with rapid turnaround time, so that providers and young people can reinforce good adherence or identify challenges in a timely manner. Finally, an electronic platform is intended to facilitate communication (e-collaboratives) and shared problem solving among providers who are often isolated in rural clinics.

To evaluate the effect of the SEARCH-Youth intervention, we used a cluster randomised clinical trial design, with clinics as the unit of randomisation. We chose this design because the implementation of the SEARCH-Youth intervention occurs at a clinic level and the risk of contamination of the control condition at the provider and participant levels is minimised. The primary objective of the trial was to establish the effectiveness of the SEARCH-Youth intervention in increasing the proportion of participants with virological suppression.

## Methods

### Study design and participants

This study was a cluster randomised, unblinded, controlled trial, with clinics as the unit of randomisation. We selected clinics in rural regions (approximately 9000–11 000 people) in western Kenya and southwestern Uganda that were operated by the government, provided antiretroviral therapy to similar numbers of young people with HIV (approximately 25–400 people), and were geographically distant from other potential trial clinics. We excluded clinics with small youth clinic patient population sizes (<25 people). Female and male individuals aged 15–24 years with HIV were recruited from local health centres and were eligible if they had a confirmed HIV diagnosis, and had received or were initiating care at a study clinic. Written informed consent was obtained before enrolment from all participants. Minors aged 15–17 years independently provided informed consent in accordance with Ugandan and Kenyan guidelines for research related to care for patients with a sexually transmitted infection. The ethical and institutional review boards of Makerere University (Kampala, Uganda), the Uganda National Council for Science and Technology, the Kenya Medical Research Institute, and the University of California San Francisco (San Francisco, CA, USA) approved this study.

### Randomisation and masking

We randomised the eligible clinics within strata of total youth clinic patient population size (<300 or ≥300 people), region (Kenya or Uganda), and previous participation in the SEARCH trial.^6^ Randomisation was done by an independent statistician using a random number generator. Clinics were not masked to the randomisation group, but the study statistician (LBB) was masked until trial completion.

### Procedures

Participants at both intervention and control clinics received the local standard clinical care, including any youth-targeted programmes being implemented by the countries’ ministries of health or other organisations (appendix p 2). Caregivers were included in care at the discretion of youth participants, assisting the study teams in contacting youth participants and supporting adherence at home. In control clinics, routine viral load monitoring was implemented every 6 months according to Ugandan and Kenyan guidelines and using ministry of health laboratory facilities.^7,8^ In the current standard of care, results are generally reported at the next routine visit, with adherence counselling for patients with a viral load higher than 1000 copies per mL and retesting during a 3-month period.

In intervention clinics, the SEARCH-Youth intervention was added to standard practices and comprised four components (appendix p 2). The life-stage assessment was done by clinicians at intervention sites using devoted tablet-based software developed by our team (appendix pp 3–4) at every routine visit. Alternative clinic access was offered as needed to address perceived barriers. In intervention clinics, routine viral load testing occurred at the same frequency as in control clinics. However, the mechanism for testing and results communication differed in intervention clinics. Plasma was transported the same or next day to one of the hubs, tested using the Xpert HIV-1 Viral Load assay (Cepheid, Sunnyvale, CA, USA) with results communicated to the clinicians by phone or electronically, who then reported results to participants by phone or in person (mechanism chosen by the participants at the previous visit) within a target turnaround time of less than 72 h. Adherence counselling was provided if the viral load was higher than 200 copies per mL, and with repeat viral testing per provider discretion as soon as 2 weeks later. Clinicians were prompted to consider e-collaboratives if they sought input on challenges such as adherence and stigma. E-collaborative discussions were done using WhatsApp, providing end-to-end encryption, and without using patient-specific information to protect the confidentiality of participants.

The COVID-19 pandemic introduced a high strain on participants and providers at our study sites during the study period, including school closures and travel restrictions (appendix p 9). The national health ministries of both countries advised HIV services to institute and rapidly scale up alternative care delivery approaches: home delivery of drugs, offsite visits, phone-based counselling, and 6 months’ drug refills; all of these services were already being implemented at intervention sites.

We trained clinicians in both control and intervention sites on the development of young individuals from age 15 years through adolescence and into adulthood and their associated medical issues (eg, contraception and alcohol use) using country-approved curricula. We additionally trained the clinicians at intervention sites in the components of the SEARCH-Youth intervention (life-stage tool, clinic access choice, rapid viral load feedback, and e-collaboratives) 2 weeks before the study start.

Data collection for study endpoints occurred through several mechanisms. Patient satisfaction was assessed using a Likert-type scale, ranging from strongly disagree to strongly agree. Care engagement, clinic transfers, and treatment changes were ascertained from clinical charts. Use of each component of the SEARCH-Youth intervention was measured using the tablet-based study form data.

For the costing analyses, we did 2-week site visits during enrolment and follow-up at both intervention and control facilities. During these visits, clinic and study staff were interviewed to identify resources expended during care for young people with HIV; implementation staff completed self-administered time-and-motion surveys to assess the proportion of their effort required to provide HIV care and implement study activities. We have previously used these methods to assess the costs of streamlined HIV care for individuals aged 15 years or older in this setting.^9^

### Outcomes

The primary outcome was the clinic-level proportion of study participants with virological suppression (HIV RNA <400 copies per mL) at 2 years of follow-up. We prespecified that the primary endpoint analysis would include data from participants enrolled before Dec 1, 2019; the rationale for this decision was to facilitate timely communication of study results and was discussed with the data and safety monitoring board on April 29, 2021. We also prespecified that people who withdrew consent, had formally transferred care, or outmigrated (defined as moving more than a 3-h travelling distance from the study clinic) would be excluded from the primary analysis; the rationale for this decision was that these people no longer represented the target population—ie, adolescents and young adults living with HIV and in the catchment area of the study clinics.

The primary analysis included all remaining participants, and among those participants, we classified their primary endpoint as either suppressed or not. Then we calculated the clinic-specific proportion of young people with virological suppression. To assess the robustness of these decisions, we prespecified sensitivity analyses to include people who outmigrated or transferred care, to exclude participants with missing endpoint viral loads, and to adjust for differences between participants with measured versus missing endpoint viral loads.

Secondary, prespecified outcomes were care engagement, switches to dolutegravir, patient satisfaction, intervention implementation, severe adverse events, and costing. These outcomes were assessed in participants who enrolled before Dec 1, 2019. Additional secondary, prespecified outcomes (ie, barriers and facilitators of the intervention, longitudinal virological suppression, alcohol use, HIV-free survival among infants born to participants, rates of vertical transmission, and mental health) will be published separately. Further details on primary and secondary outcomes are available in the statistical analysis plan (appendix p 10).

### Statistical analysis

Using sample size formulas for cluster randomised trials with proportion endpoints,^10^ we estimated that 28 clinics (14 clinics per group) would provide 80% power to detect at least a 24% relative increase in virological suppression at 2 years of follow-up from 65% in the control, assuming a coefficient of variation of k=0·175 and a harmonic mean of 50 participants per clinic.

In the primary analysis, we compared the average proportion of young people with virological suppression at 2 years of follow-up with targeted minimum loss-based estimation, an approach that accounts for the dependence of outcomes within clusters and adaptively adjusts for baseline covariates to maximise precision.^11^ Specifically, we used leave-one-out cross-validation to select from the following prespecified candidate adjustment variables: the clinic-specific number of adolescents and young adults in HIV care at baseline, the clinic-specific proportion of young people with virological suppression among those engaged at baseline, or no adjustment. Using the Student’s *t*-distribution, we calculated two-sided 95% CIs and tested the null hypothesis that the SEARCH-Youth intervention did not improve virological suppression compared with the control intervention, with a one-sided test at the 5% significance level.

Prespecified subgroups included sex, age group (15–19 years and 20–24 years), and baseline care status (recently engaged [started treatment within 6 months of or at enrolment], engaged [started treatment more than 6 months before enrolment and had a clinic visit within 6 months before enrolment], and re-engaging [started treatment more than 6 months before enrolment and without a clinic visit within 6 months of enrolment]). Prespecified sensitivity and secondary analyses, including the impact of transition to dolutegravir-based regimens, are described in the statistical analysis plan (appendix p 10).^12^ All analyses were done in R, version 4.0.3.

For costing, we entered data into a standardised Excel workbook and analysed the data to estimate the incremental annual cost per participant associated with SEARCH-Youth activities. Time-and-motion data were used to estimate the proportion of active work time that was dedicated to HIV care and study activities for each staff cadre in each facility. These data were included in the micro-costing to estimate the direct and indirect costs of patient visits in intervention and control clinics. Annual costs for each patient assume three clinic visits per year, US$65 per patient per year for a dolutegravir-based antiretroviral treatment regimen, and an annual viral load test ($31·48 for a rapid viral load test in intervention facilities and $110 for a traditional viral load test in control facilities). This trial is registered with ClinicalTrials.gov, NCT03848728.

### Role of the funding source

The funder of the study had no role in study design, data collection, data analysis, data interpretation, or writing of the report.

## Results

**Figure 1:**
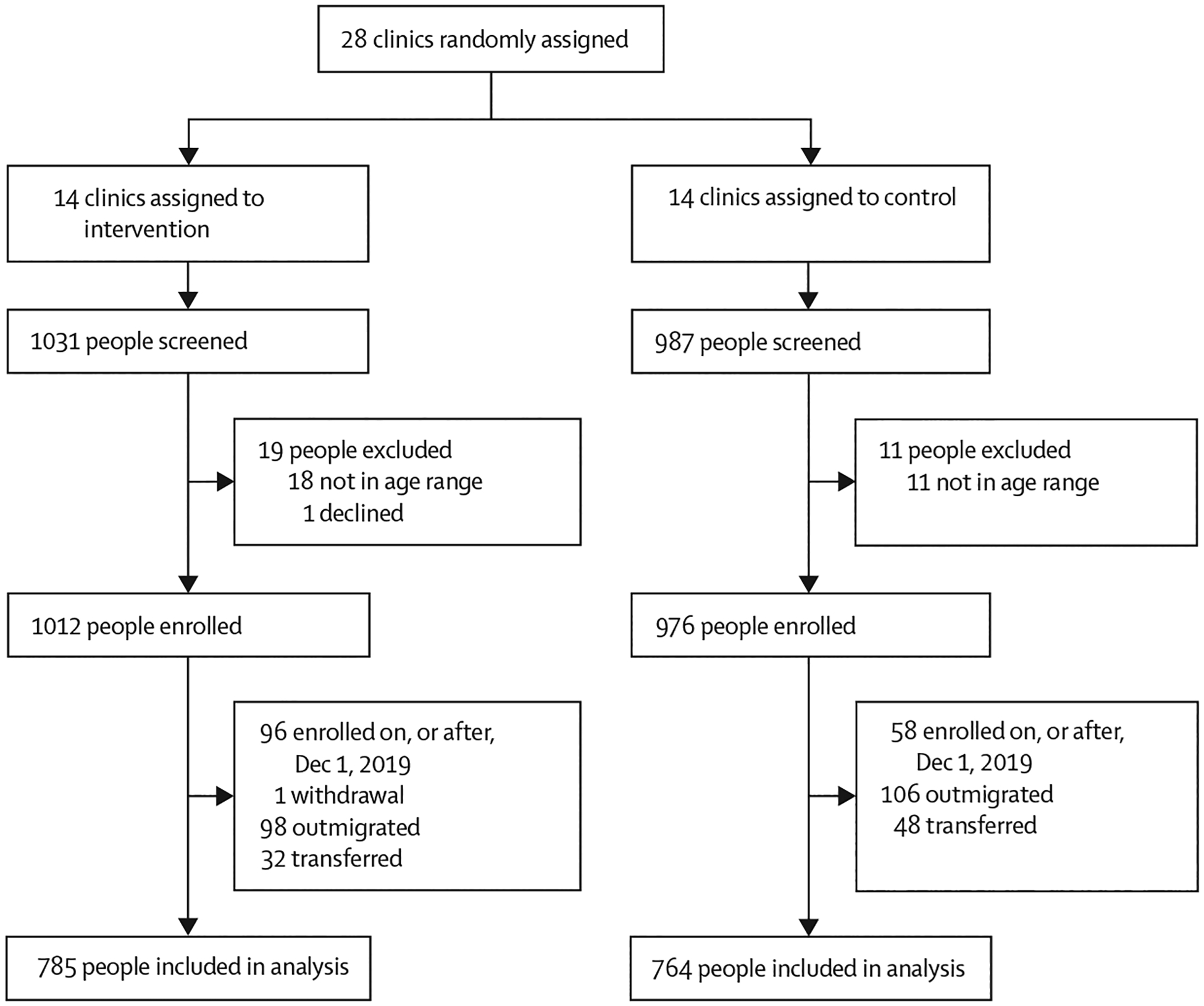
Trial profile

In January, 2019, we included 28 clinics and excluded four due to their small patient population sizes. The eligible clinics were randomly assigned: 14 were assigned to the intervention and 14 to the control, balanced on country (14 in Kenya and 14 in Uganda). From March 14, 2019, to Nov 26, 2020, we recruited 1988 participants, of whom 1834 had enrolled before Dec 1, 2019: 916 (90·5%) of 1012 participants at intervention clinics and 918 (94·1%) of 976 participants at control clinics (figure 1), who were followed up until March 1, 2022. During the 2-year follow-up, one participant at an intervention clinic withdrew consent. Between groups, similar proportions of participants outmigrated (98 [10·7%] of 915 participants in intervention clinics *vs* 106 [11·5%] of 918 participants in control clinics) or transferred care (32 [3·5%] *vs* 48 [5·2%]). All clusters were included in the analysis. The remaining 1549 participants (785 [50·7%] participants in intervention clinics and 764 [49·3%] participants in control clinics) comprised the prespecified analytical cohort for the primary endpoint.

Participants’ median age was 21 years (IQR 19–23) and 1248 (80·6%) of 1549 participants were female (table 1). Most participants were already engaged in care at the time of enrolment (1026 [66·3%] of 1547), with similar distribution of care status within groups. More female participants (47 [3·8%] of 1246) were re-engaging in care at baseline than male participants (eight [2·7%] of 301). The most common antiretroviral regimen at enrolment was tenofovir, lamivudine, and efavirenz (1118 [72·9%] of 1533). The overall proportion of participants with virological suppression at baseline was 74·8% (1147 of 1533): 73·5% (573 of 780) in intervention clinics and 76·2% (574 of 753) in control clinics. Baseline characteristics of the entire cohort (appendix pp 5–6) were similar to that of the analytical cohort (table 1). Baseline characteristics at the clinic level are provided in the appendix (p 6).

Endpoint viral loads were obtained in 1425 (92·0%) of 1549 participants: 731 (93·1%) of 785 in intervention clinics and 694 (90·8%) of 764 in control clinics in the analytical cohort. 17 (1·1%) deaths occurred: eight (1·0%) in intervention clinics and nine (1·2%) in control clinics. The remaining 107 (6·9%) participants did not have their endpoint viral load measured: 46 (5·9%) in intervention clinics and 61 (8·0%) in control clinics. No severe adverse events thought to be associated with the intervention were reported.

In the primary analysis, the mean proportion of participants with virological suppression at 2 years of follow-up was 88% (95% CI 85–92) in intervention clinics and 80% (77–84) in control clinics, corresponding to a 10% beneficial effect from the SEARCH-Youth intervention (risk ratio [RR] 1·10, 95% CI 1·03–1·16; p=0·0019; appendix p 6). Similar results were observed in the prespecified sensitivity analyses including participants who outmigrated or transferred care (1·12, 1·04–1·20; p=0·0016), excluding participants with missing endpoints (1·06, 1·01–1·12; p=0·0076), and adjusting for differences in characteristics between people with measured versus missing endpoints (1·08, 1·02–1·13; p=0·0036; appendix p 6).

**Figure 2:**
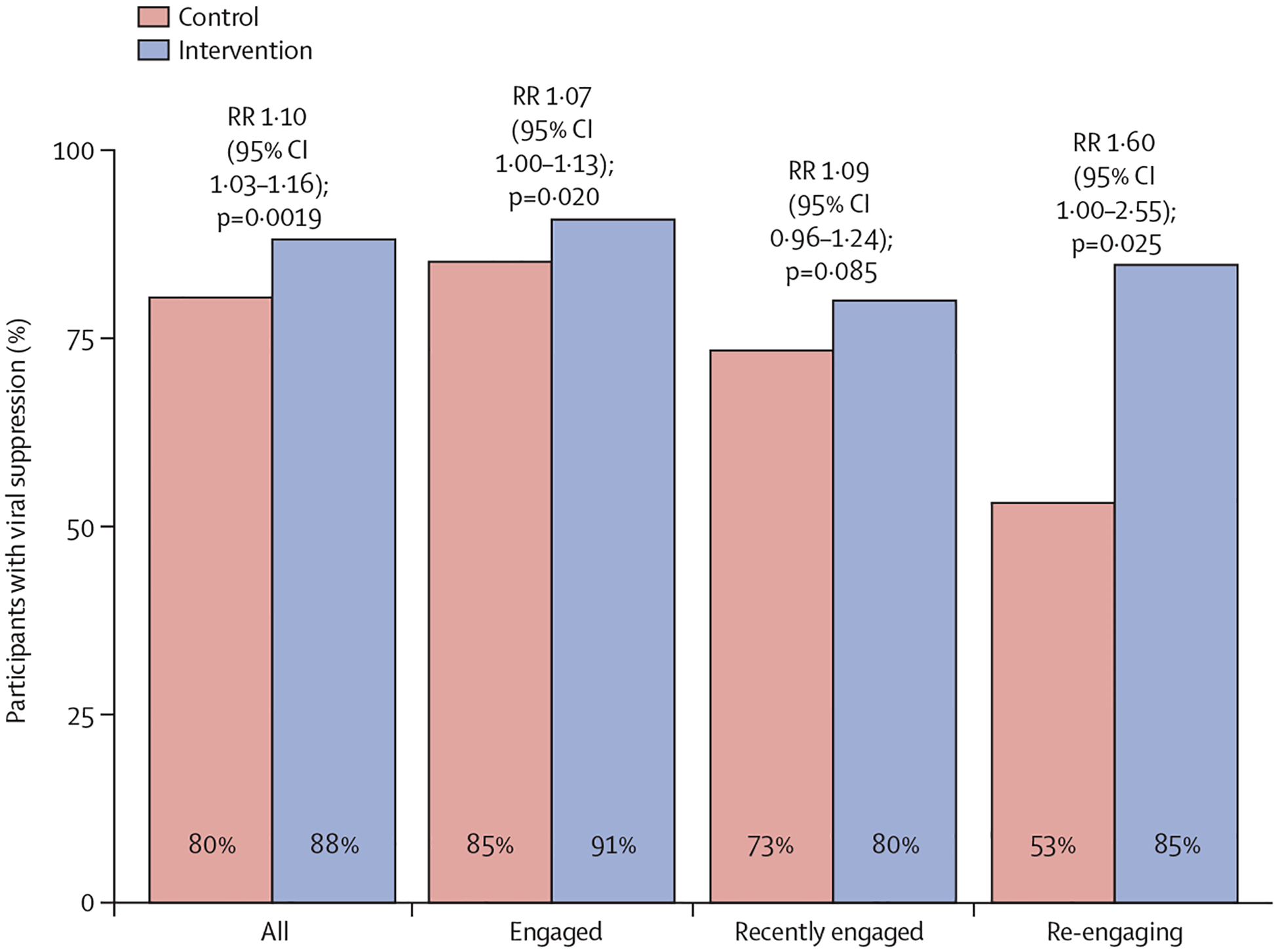
Proportion of participants with virological suppression at 2 years of follow-up, stratified by baseline care status RR=risk ratio.

Across the prespecified subgroups, the intervention was beneficial but varied in effect size. Among strata of HIV care at baseline, the greatest benefit was among participants re-engaging in care (RR 1·60, 95% CI 1·00–2·55; p=0·025; figure 2). The SEARCH-Youth intervention increased virological suppression in both female participants (1·06, 1·00–1·13; p=0·026) and male participants (1·11, 0·98–1·25; p=0·044), as well as in older (aged 20–24 years) participants (1·05, 0·99–1·11; p=0·040) and younger (aged 15–19 years) participants (1·13, 1·01–1·26; p=0·0015; appendix p 7).

**Figure 3:**
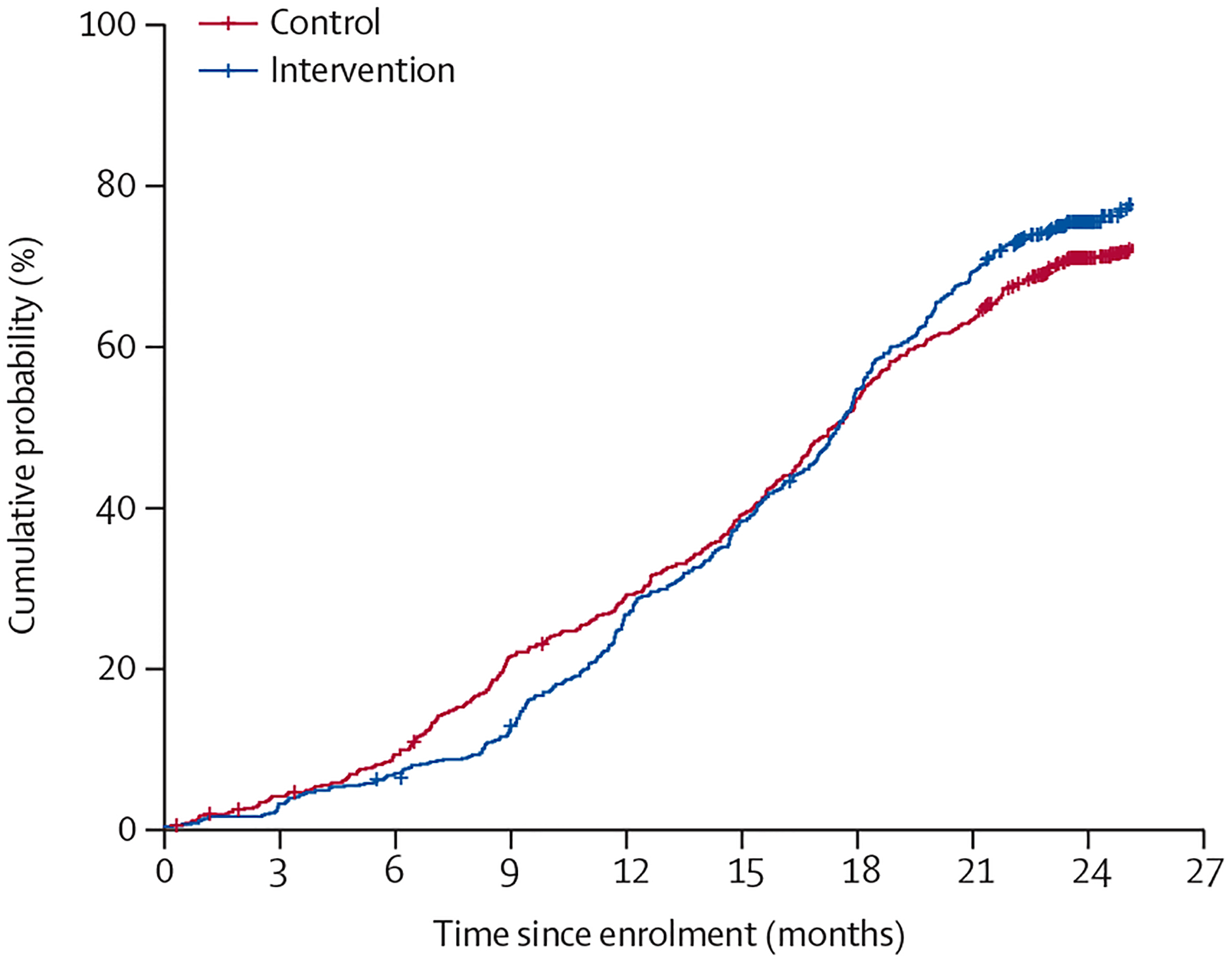
Cumulative probability of switching to a dolutegravir-based treatment

During the 2-year study period, 1003 (64·8%) of 1549 participants switched to an antiretroviral combination regimen that included dolutegravir, with a higher proportion of participants in the intervention group (77%, 95% CI 73–82) than in the control group (71%, 66–75; RR 1·10, 95% CI 1·00–1·20; p=0·041; figure 3). The intervention had a beneficial effect on virological suppression in both participants who had switched to a dolutegravir-based treatment and those who did not, although it did not reach statistical significance when stratified (table 2). The joint probability of switching to a dolutegravir-based treatment and attaining virological suppression by 2 years was also 11% higher in participants in the intervention group (70%, 95% CI 65–75) than in those in the control group (63%, 59–67; RR 1·11, 95% CI 1·00–1·22; p=0·020).

The SEARCH-Youth intervention also increased retention in HIV care. Specifically, the proportion of young people who had contact with the clinic in the previous 6 months to endpoint ascertainment was 91% (95% CI 89–94) among participants in intervention clinics and 72% (61–82) among participants in control clinics, corresponding to a 27% increase (RR 1·27, 95% CI 1·10–1·47; p=0·0010).

Patient satisfaction surveys were completed by 716 (91·2%) of 785 participants in intervention clinics and 713 (93·3%) of 764 participants in control clinics. The intervention was associated with increased trust that the provider would “keep my information private”, a perception of ease in the ability to “get in touch with my provider”, and a sense that providers knew “how to treat young people with HIV” (table 3).

In the intervention group, there was a high use of the life-stage assessment. Specifically, 663 (84·5%) of 785 participants had at least four assessments. Offsite appointments, phone visits, out-of-hours appointments, and offsite drug delivery were selected by many participants, and access choice options varied by clinic (appendix p 7). Across the 14 intervention clinics, the median proportion of viral load results delivered within 72 h was 95%, and the median delivery time was 1·35 days (appendix p 8). Providers used the e-collaborative platform for 262 discussions about 127 study participants with challenging management issues.

To estimate the cost of HIV care and intervention activities, we did 2-week site visits in 14 facilities during enrolment (seven intervention and seven control facilities, evenly distributed between Kenya and Uganda) and 23 facilities during follow-up (15 intervention and eight control facilities, evenly distributed between countries). The annual cost of HIV care per patient was US$7·71 more expensive in intervention facilities than in control facilities ($269·68 and $261·97, respectively; appendix p 8). The intervention was associated with higher costs per patient annually in recurrent goods ($43·05 higher), personnel costs ($24·97 higher), facility costs ($5·17 higher), and capital goods (the equipment needed for Xpert viral load testing; $0·31 higher). These additional costs were partly offset by lower services costs ($65·79 lower), primarily due to lower costs associated with rapid viral load testing compared with the standard practice of sending blood plasma samples to central laboratories for testing.

## Discussion

88% of adolescents and young adults living with HIV and participating in the SEARCH-Youth intervention had virological suppression at 2 years, representing an improvement compared with standard care (80%). Although there has been great progress in HIV treatment coverage globally, improvements in clinical outcomes for adolescents and young adults have lagged behind those for older adults.^2^ The gains in care engagement and virological suppression derived from the SEARCH-Youth intervention brought adolescents and young adults with HIV in this cohort closer to the UNAIDS 2030 95-95-95 targets.

The development of interventions to improve outcomes among adolescents and young adults with HIV in sub-Saharan African has been slow, but some approaches are now showing success. A 2019 review of interventions to improve antiretroviral therapy adherence among adolescents and young people identified reports of three patient-level interventions and four health services interventions, but none were shown to increase virological suppression.^13^ Implementation of the Kenyan Adolescent Package of Care did not increase virological suppression among adolescents in one study;^14^ however, another retrospective study found that the presence of youth-friendly services plus trained providers was associated with increased rates of virological suppression at clinics.^15^ Peer-based strategies are now recommended by WHO and show promising results.^16^ The Baylor College of Medicine International Pediatric AIDS Initiative Teen Clubs increased retention in a case-control study of adolescents in Malawi.^17^ In Project YES!, a youth peer-mentor programme in clinics resulted in increased virological suppression in young individuals aged 15–24 years.^18^ There is also increasing evidence showing benefit from combination service delivery approaches, as recommended by UNAIDS.^19^ The Zvandiri programme combined community adolescent treatment supporters and monthly support groups with text messaging, calls, home visits, and clinic-based counselling; in a cluster randomised trial, fewer participants in the Zvandiri intervention group (52 [25%] of 209) had non-virological suppression (>1000 copies per mL) or died than participants in the standard HIV care group (97 [36%] of 270; p=0·03).^20^

The SEARCH-Youth intervention is distinct from other care delivery models by combining elements at the levels of client–provider relationships (life-stage-based discussion) and facility (offering out-of-hours, offsite, and phone access appointments) with a biomedical tool (rapid viral load feedback) and a communication platform to facilitate collaboration among providers at different rural facilities. The intervention was designed to add resilience against pressures on adherence and care, and to be dynamic in identifying and adapting to new issues over time. The COVID-19 pandemic introduced high external strain on participants in our study in many ways, including restrictions on travel implemented by the Governments of Kenya and Uganda (appendix p 9). One adult clinical programme in Uganda reported a 46% decrease in clinic visits overall in 2020 compared with the previous year, with 42% of participants (n=14 632) reporting difficulty in travelling to the clinic.^21^ Additionally, there has been particular concern about the impact of the COVID-19 pandemic on adolescents living with HIV globally. The SEARCH-Youth intervention showed benefit even within the constraints of the COVID-19 pandemic and when many of the access components of the intervention (eg, phone-based counselling and 6 months drug refills) were being recommended by the ministries of health across all sites. It is possible that due to our pre-existing phone-based activities, intervention clinicians could more easily reach patients and help them arrange alternate drug pick-up away from their usual providers while counselling and life-stage assessment were done by phone.

The life-stage tool was intended to create a collaborative relationship between providers and clients, helping them identify and anticipate barriers to adherence and care that can shift through this period of life. At the start of this trial, we examined the prevalence of recent life-stage events in the intervention group using data from the first life-stage assessment and found that two or more major life-stage events (eg, changes in schooling, employment, or partnerships, or becoming a parent) were associated with no virological suppression.^22^ The tool was also designed to help providers elicit and address issues of stigma with individualised solutions such as alternative access options (eg, coming to the clinic at early or late hours to accommodate school or work schedules). Stigma at individual, interpersonal, community, and organisational levels intersects with and continues to produce substantial barriers to treatment among young people living with HIV in Africa. Qualitative study of our participant population at baseline suggested that non-disclosure of HIV status, medication schedules, and clinic appointments all elicited stigma at school.^23^ The results of our patient satisfaction survey suggest that the intervention succeeded in increasing trust in providers to keep information private and that convenience in alternative visit times was appreciated.

Providers in intervention clinics were very successful in implementing rapid viral load feedback, with a median of 95% of viral load results delivered to clients within 72 h (appendix p 8). Point-of-care viral load assays with rapid turnaround times are now being implemented more widely in low-income and middle-income countries, but outcomes have been mixed. In a randomised controlled trial, point-of-care viral load testing every 3 months for Kenyan children younger than 14 years was not associated with increased prevalence of virological suppression.^24^ However, point-of-care viral load testing was associated with increased virological suppression in a study of South African adults.^25^ We postulated that the rapid feedback of viral loads—in both supporting successful adherence and identifying lapses—had the potential to be especially effective for adolescents and young people who are just developing the cognitive skills of abstract thinking and appreciating long-term consequences of actions.^3^ However, we could not discern the specific contribution of the rapid viral load feedback within the combination SEARCH-Youth model of care.

A key component of the success of the SEARCH-Youth intervention was the higher rate of retention in care in intervention clinics (91%) compared with control clinics (72%). One of the main drivers of poor outcomes in young people living with HIV in sub-Saharan Africa is disengagement from care, especially among adolescents. A systematic review estimated that more than 15% of adolescents are lost to follow-up across sub-Saharan Africa,^26^ but rates as high as 52% in South Africa^27^ and 32% in rural Uganda^28^ have been reported. We postulate that a perception of satisfaction with care is important in maintaining clients in care. Overall patient satisfaction has been associated with re-engagement with HIV care in adults.^29^ We found that participants in the intervention group were much more satisfied with care than participants in the control group (table 3).

Several features of the design and timing of this trial contribute to the generalisability of our results to the current treatment landscape for young people and their utility to policy makers. We used a pragmatic^30^ design that included minimal enrolment criteria and clinic-based recruitment to better approximate real-world implementation. We built flexibility into the intervention (eg, alternative access options varying by participant), did not disrupt participant access to other local programmes, and generally aligned with standard country follow-up schedules. The timing of this trial, during the roll-out of dolutegravir-based regimens in these regions, also increases its relevance to the current context. During the 2-year study period, both Kenya and Uganda transitioned to tenofovir–lamivudine–dolutegravir for people living with HIV, as per WHO guidance.^31^ Evidence from clinical programmes of adults and clinical trials in adolescents suggest that dolutegravir-based regimens result in improved rates of virological suppression.^32^ We found that the SEARCH-Youth intervention facilitated the transition of young people to tenofovir–lamivudine–dolutegravir, with a higher proportion of intervention participants having switched by 2 years (figure 3). Transition to dolutegravir was probably a key driver to the high rates of virological suppression in the control group (80%). Importantly, we also found that the intervention appeared to confer benefit not just to participants who switched to dolutegravir-based treatment, but also among those who had not switched (table 2).

Increasing evidence including from studies done in Uganda shows that rapid viral load feedback is feasible to implement at scale. A cluster randomised trial in 20 clinics in Uganda showed that rapid viral testing using the GeneXpert was feasible, with median turnaround time of 1 day.^33^ The Ugandan Ministry of Health is currently prioritising improvements in turnaround time for viral load results and moving to communicate results to clinics electronically; clinics in Uganda are also implementing same-day point-of-care viral load testing with the GeneXpert assay in their follow-up programme for infants born to women with HIV. An additional consideration in implementing this intervention on a large scale would be its cost. We estimated that the SEARCH-Youth intervention was associated with a modest increase of $7·71 (3%) in annual cost per patient. If point-of-care viral load testing became standard of care, then our intervention would be associated with $30·35 (12%) in additional costs per patient per year. These costs are lower than what has been observed in several studies of non-clinical interventions for adolescents and young adults with HIV, ranging from $49·50 to $166·02 in additional costs per patient per year.^34,35^

Our study had limitations. As is characteristic for this age group, our participants had a high degree of mobility, resulting in transfers of care and outmigration. To address this shortcoming, we tracked all participants who had outmigrated or transferred care and included them in a secondary analysis that also showed benefit of the intervention among those who had separated from the study community. Another limitation of our study is the challenge of identifying which elements of our multicomponent intervention had the greatest benefit. Ongoing qualitative studies of providers and participants might provide insight into this aspect. Our costing estimates were limited to the ongoing costs of HIV care for adolescents and young adults. Programmes implementing this intervention would incur additional costs associated with training of personnel. The implication of these costs on the additional cost per person would depend on the number of adolescents and young adults served by this intervention.

As they transition into adulthood, adolescents and young adults living with HIV in sub-Saharan Africa face numerous challenges to remaining in care and having virological suppression. Dynamic models of care that accommodate their individual needs and increase resilience in their relationships with providers and clinics are needed. The multilevel SEARCH-Youth intervention improved outcomes among young people when added to standard practices, providing benefits beyond transition to dolutegravir, and was effective despite COVID-19 pandemic-related restrictions. Systematic life-stage-based assessment and similar multilevel flexible support could help bring adolescents and young adults living with HIV closer towards a goal of universal virological suppression.

## Supplementary Material

Supplementary Material

## Figures and Tables

**Table 1:** Baseline characteristics of study participants in the analytical cohort

	Intervention (n=785)	Control (n=764)	Total (n=1549)
Age, years	21 (19–23)	22 (19–23)	21 (19–23)
Sex			
Female	643 (81·9%)	605 (79·2%)	1248 (80·6%)
Male	142 (18·1%)	159 (20·8%)	301 (19·4%)
Country of residence			
Kenya	332 (42·3%)	324 (42·4%)	656 (42·3%)
Uganda	453 (57·7%)	440 (57·6%)	893 (57·7%)
Education (started or completed)			
No school	31 (3·9%)	24 (3·1%)	55 (3·6%)
Primary school	507 (64·6%)	525 (68·7%)	1032 (66·6%)
Secondary school	194 (24·7%)	175 (22·9%)	369 (23·8%)
Tertiary school	53 (6·8%)	40 (5·2%)	93 (6·0%)
At boarding school	56 (7·1%)	56 (7·3%)	112 (7·2%)
Employment status			
Employed	283 (36·1%)	308 (40·3%)	591 (38·2%)
In school	165 (21·0%)	155 (20·3%)	320 (20.7%)
Unemployed	337 (42·9%)	301 (39·4%)	638 (41·2%)
Marital status			
Single, never married	342 (43·6%)	291 (38·1%)	633 (40·9%)
Married, monogamous	306 (39·0%)	327 (42·8%)	633 (40·9%)
Married, polygamous	44 (5·6%)	51 (6·7%)	95 (6·1%)
Widowed	3 (0·4%)	6 (0·8%)	9 (0·6%)
Divorced	90 (11·5%)	89 (11·6%)	179 (11·6%)
Number of children			
0	330 (42·0%)	323 (42·3%)	653 (42·2%)
1	258 (32·9%)	227 (29·7%)	485 (31·3%)
2	129 (16·4%)	131 (17·1%)	260 (16·8%)
3–5	51 (6·5%)	64 (8·4%)	115 (7·4%)
Drinks alcohol	137 (17·5%)	94 (12·3%)	231 (14·9%)
Mobile*	210 (26·8%)	229 (30·0%)	439 (28·3%)
Antiretroviral regimen at enrolment†			
TDF–3TC–EFV	591 (76·0%)	527 (69·8%)	1118 (72·9%)
TDF–3TC–DTG	81 (10·4%)	101 (13·4%)	182 (11·9%)
AZT–3TC–NVP	33 (4·2%)	42 (5·6%)	75 (4·9%)
TDF–3TC–ATV/r	10 (1·3%)	18 (2·4%)	28 (1·8%)
AZT–3TC–ATV/r	12 (1·5%)	14 (1·9%)	26 (1·7%)
ABC–3TC–EFV	12 (1·5%)	11 (1·5%)	23 (1·5%)
Other	39 (5·0%)	42 (5·6%)	81 (5·3%)
Baseline viral load <400 copies per mL‡	573 (73·5%)	574 (76·2%)	1147 (74·8%)
Baseline care status§			
Recently engaged¶	252 (32·1%)	214 (28·0%)	466 (30·1%)
Engaged∥	496 (63·3%)	530 (69·5%)	1026 (66·3%)
Re-engaging**	36 (4·6%)	19 (2·5%)	55 (3·6%)

Data are n (%) or median (IQR). Data are restricted to the analytical population of participants who enrolled before Dec 1, 2019, and who did not withdraw from the study, transfer, or outmigrate. 3TC=lamivudine. ABC=abacavir. ATV/r=ritonavir-boosted atazanavir. AZT=zidovudine. DTG=dolutegravir. EFV=efavirenz. NVP=nevirapine. TDF=tenofovir disoproxil fumarate.

* Lived away from home for more than 1 month in the previous 6 months.

† Data missing for 16 (1·0%) participants: seven (0·9%) in the intervention group and nine (1·2%) in the control group.

‡ Data missing for 16 (1·0%) participants: five (0·6%) in the intervention group and 11 (1·4%) in the control group.

§ Data missing for two (0·1%) participants: one (0·1%) in the intervention group and one (0·1%) in the control group.

¶ Started treatment within 6 months of enrolment or at enrolment.

∥ Started treatment more than 6 months before enrolment and had an HIV care visit within 6 months of enrolment.

** Started treatment more than 6 months before enrolment, but did not have an HIV care visit within 6 months of enrolment.

**Table 2:** Proportion of individuals with virological suppression among participants who switched and did not switch to dolutegravir-based treatment

	Intervention (95% CI)	Control (95% CI)	Effect of intervention (95% CI)*	p value
Switched	92% (89–95)	88% (84–92)	1·04 (0·99–1·10)	0·057
Did not switch	70% (61–79)	64% (54–74)	1·09 (0·89–1·34)	0·19

* Relative scale using two-stage targeted minimum loss-based estimation.

**Table 3:** Differences in patient satisfaction in the intervention and control groups

	Effect of intervention (95% CI)*	p value
“The time and date of my appointments are convenient for me.”	0·34 (0·07 to 0·60)	0·0069
“It is easy to get in touch with my provider.”	0·36 (0·11 to 0·61)	0·0029
“Phone visits are (or would be) convenient for me.”	−0·04 (−0·32 to 0·24)	0·61
“I like drug delivery outside of the clinic at a location convenient for me.”	046 (0·04 to 0·89)	0·017
“I trust my provider will keep my information private.”	0·30 (0·1 to 0·49)	0·0020
“My provider knows how to treat young people with HIV.”	0·31 (0·1 to 0·53)	0·0028
“The staff at this clinic care about me.”	0·21 (−0·06 to 0·48)	0·064
“I would recommend this clinic to other young people living with HIV.”	0·25 (0·02 to 0·47)	0·018
“Overall, I feel satisfied with the care I receive at this clinic.”	0·30 (0·05 to 0·54)	0·011

* Difference scale using two-stage targeted minimum loss-based estimation.

## Data Availability

De-identified study data will be made available approximately 1 year after completion of the ongoing trial (NCT03848728), following approval of a concept sheet summarising the analyses to be done. Further inquiries can be directed to the SEARCH Scientific Committee at douglas.black@ucsf.edu.

## Research in context

### Evidence before this study

Adolescents and young adults living with HIV have lower rates of virological suppression than older adults, and few interventions have shown evidence of improving outcomes. We searched PubMed for articles published in English from database inception to Sept 28, 2022, using the terms: “(HIV) AND ((adolescents) OR (youth) OR (young adults)) AND ((virologic) or (viral)) and (suppression) and (Africa)”. We identified 834 articles. Before this study, a systematic review of articles published from 2015 to 2019 about interventions to improve antiretroviral therapy adherence among adolescents and young people in low-income and middle-income countries identified only three patient-level interventions and four health services interventions, none of which increased rates of virological suppression.

### Added value of this study

The SEARCH-Youth intervention was designed to provide dynamic support to adolescents and young adults with HIV in rural sub-Saharan Africa as they developed into adults, facing potentially disruptive events. The intervention combined a life-stage-based assessment to help providers and patients identify potential challenges to retention and adherence, with alternative options for clinic access to address barriers, rapid viral load testing to provide timely feedback about adherence, and a WhatsApp platform to promote collaboration among isolated rural providers. In a pragmatic cluster randomised trial, the SEARCH-Youth intervention resulted in higher rates of virological suppression and retention in care at 2 years of follow-up. During the study period, the study regions transitioned young individuals to dolutegravir-based treatment and were subject to mandated closures from the COVID-19 pandemic. Our results represent rigorous evidence in support of a new, multilevel service delivery model, including interventions at patient, provider, and clinic levels, which improves the virological outcomes of adolescents and young adults living with HIV, generalisable to the current treatment context in rural sub-Saharan Africa.

### Implications of all the available evidence

Our study adds to a growing evidence base that we can increase virological suppression with multicomponent interventions in young people living with HIV. Recent trials have shown that combinations of peer mentoring with facilitated transitions to adult care, and peer-led differentiated service delivery with adherence support can result in increased virological suppression. Interventions such as SEARCH-Youth, applying life-event informed multilevel dynamic support to adolescents and young adults with HIV and providers, should be used to achieve the ultimate goal of universal virological suppression across all ages.
