# Supplementary material for: A multilevel health system intervention for virological suppression in adolescents and young adults living with HIV in rural Kenya and Uganda (SEARCH-Youth): a cluster randomised trial
Source: Lancet HIV. Author manuscript; Available in PMC 2024 Jun 7. (PMC11158418; doi:10.1016/S2352-3018(23)00118-2)
Supplement: Supplementary Material [file NIHMS1998338-supplement-Supplementary_Material.pdf]

THE LANCET
HIV

Supplementary appendix

This appendix formed part of the original submission and has been peer reviewed. We post it as supplied by the authors.

Supplement to: Ruel T, Mwangwa F, Balzer LB, et al. A multilevel health system intervention for virological suppression in adolescents and young adults living with HIV in rural Kenya and Uganda (SEARCH-Youth): a cluster randomised trial. *Lancet HIV* 2023; **10:** 518–27.

Appendix

Supplementary Material

Title: SEARCH-Youth: a cluster randomized trial of a multilevel health system intervention to improve virologic suppression in adolescents and young adults living with HIV in rural Kenya and Uganda

Table of Contents

Table S1. SEARCH Youth Intervention Components Page 2

Table S2. Youth focused activities available in study Page 2

Table S3. SEARCH Youth life stage assessment Page 3

Table S4. Characteristics of study participants at enrolment, by arm and overall Page 5

Table S5. Clinic-level baseline characteristics, by arm and overall. Page 6

Table S6. Arm-specific and intervention effect estimates on virologic suppression when varying the approach to defining the analytic population and analytic approach to missingness Page 6

Table S7. Arm-specific and intervention effect estimates on virologic suppression by baseline demographic group Page 7

Figure S1. Alternative access choice among intervention participants, by clinic Page 7

Table S8. Completion and timing of viral load results delivery Page 8

Table S9. Annual Cost per Adolescent or Young Adult Page 8

Figure S2. Timeline of COVID-19 restrictions affecting COVID-19 clinics during the study period Page 9

Statistical Analysis Plan Page 10

Table S10. CONSORT 2010 checklist of information to include when reporting a cluster randomised trial Page 11

References Page 14

Table S1. Youth focused activities available in study area

Country	Programs at study clinics
Kenya	Youth clubs, youth only clinic days, youth friendly activities, peer educators and adherence counseling, treatment buddies, appointment reminders, tracking, home visits, transport reimbursement, school fees, nutritional support.
Uganda	Youth clubs, youth only clinic days, youth friendly activities, peer educators, appointment reminders, tracking, home visits, transport reimbursement, school fees, nutritional support, technical skill training, off site drug delivery.

Table S2. SEARCH Youth Intervention Components

Component	Content	Frequency
Life Stage Assessment Tool	Checklist-driven structured evaluation of life issues to guide the discussion that starts each visit, including employment or educational status, major life events, social support systems, behaviours (e.g alcohol use), and HIV-disclosure status.	At every routine visit, at least twice annually
Alternative Clinic Access	Before or after hours, off site, or by telephone.	As needed at the end of each visit to address barriers
Rapid Viral Load Feedback	Provided to participant as soon as possible, with target <72 hours	At least twice annually, and more frequent if need for follow up
Provider E-collaborative	Discussion of difficult cases initiated by individual providers	As needed for difficult cases

Table S3. SEARCH Youth life stage assessment

**Location of Visit**	□ Clinic □ Offsite □ Phone If clinic, peer navigator is in clinic today: □ Yes □ No	
	Assessment	***Action***
**Life Stage**	**School or work?** □ School □ Work □ Neither □ Both<br>**Relationships**: □ Married □ In other sexual relationship □ Single	□ Counseled on family planning options<br>□ Reproductive health appointment<br>□ Other ________________________________
	**Additional details on school/work, relationships or other issues**<br>____<br>____<br>____<br>____	□ Other actions _________________________
**Life Events**	**Any major life events since last visit?**<br>□ Start or stop school or employment □ Change in residence<br>□ Divorce, separation or relationship strife □ New sexual partner<br>□ Family death □ Sickness □ Incarceration □ Family strife<br>□ Birth or pregnancy<br>*Describe key details below.*<br>____<br>____<br>____<br>____	□ Counseled about _______________________<br>□ Other _________________________________
**Support and Disclosures**	**Do you feel supported by people around you?** □ Yes □ No<br>Who are supportive persons? □ Parent/Guardian □ Partner<br>□ Friend □ Other: ______________________________<br>*Feeling lonely or isolated? Describe key details below.*<br>____<br>____<br>____<br>____	□ Discussed sources of support<br>□ Referred for peer support linkage<br>□ Other _________________________________
	**Does anyone ever hurt or threaten you?** □ Yes □ No<br>If yes, who? □ Parent/Guardian □ Partner<br>□ Other: ____<br><br>**Do you feel pressured to have sexual activity**? □ Yes □ No<br>If so, who pressures you? ______________________________<br>*Describe key details below.*<br>____<br>____<br>____	□ Actions _________________________
	**Completed and desired HIV disclosures**:<br>**Family?** □ 1 or more members □ No family members<br>**Partner?** □ 1 or more partners □ No partners<br>**Friends?** □ 1 or more friends □ No friends<br>**Other disclosures?**<br>*Describe*: ____<br>____<br>____<br>____	□ Discussed disclosure challenges and benefits<br>□ Scheduled facilitated disclosure appointment<br>□ Other ________________________________
**Mental Health/Behaviors**	How much **alcohol** do you drink?<br>Do you use any **other substances** (e.g. marijuana)?<br>How are you **protecting yourself** from sexually transmitted infections?<br>*Describe key details below.*<br>____<br>____<br>____<br>____	□ Offered condoms<br>□ Counseled about _______________________<br>□ Other _________________________________

	**Over the last 2 weeks have you felt lonely, disliked yourself, or lost hope?** □ Yes □ No<br>If yes, have you considered hurting yourself? □ Yes □ No<br>*Describe key details below.*<br>____<br>____<br>____<br>____	□ Counseled, reviewed support system<br>□ Other ______________________________
**Resources**	**How many meals did you have yesterday?**<br>(number) ________ Is that normal? □ Yes □ No<br>*Describe key details below.*<br>____<br>____<br>____<br>____	□ Discussed circumstances<br>□ Other ______________________________
	**Do you have trouble getting to the clinic for your appointments?**<br>*Describe key details below.*<br>____<br>____<br>____	□ Discussed circumstances<br>□ Other ______________________________
**Priority Issues**	Of all that we have discussed today, what do you think **most affect ability to take medicine or come to clinic**?<br><br>1.____<br>□ Affects clinic attendance □ Affects medicine adherence<br><br>2.____<br>□ Affects clinic attendance □ Affects medicine adherence<br><br>3.____<br>□ Affects clinic attendance □ Affects medicine adherence<br><br>4.____<br>□ Affects clinic attendance □ Affects medicine adherence<br><br>5.____<br>□ Affects clinic attendance □ Affects medicine adherence	**Specific Action Plans**<br><br>1.__________________________________<br><br>2.__________________________________<br><br>3.__________________________________<br><br>4.__________________________________<br><br>5.__________________________________

Access Choice for Next Routine Visit	
**Next Routine Appointment**	**If barrier identified, offered** *(tick all that apply)*:<br>□ Off hours visit: Time: ________________<br>□ Offsite visit: Location: ___________________________<br>□ Phone call visit (if suppressed)<br>□ Alternate medication delivery
	Best way to contact patient:________________________________<br>Current phone number: ____________________________________<br>Date of next routine visit: (*in standard 3 months or > 3 months if barriers to frequent visits)*<br><br>|___|___|/|___|___|/|___|___| *day month year*

Table S4. Characteristics of study participants at enrolment (including withdrawals transfers and outmigrants), by arm and overall

Characteristics	Number of Participants (%) (Total = 1834)		
	Intervention (n=916)	Control (n=918)	Total (n=1834)
**Age** (median, [Q1,Q3])	21 [19,23]	22 [19,23]	21 [19,23]
**Female**	761 (83·1%)	746 (81·3%)	1507 (82·2%)
**Country of Residence**			
-Kenya	399 (43·6%)	389 (42·4%)	788 (43%)
-Uganda	517 (56·4%)	529 (57·6%)	1046 (57%)
**Education**			
-No school	35 (3·8%)	33 (3·6%)	68 (3·7%)
-Primary School	595 (65%)	620 (67·5%)	1215 (66·2%)
-Secondary School	228 (24·9%)	219 (23·9%)	447 (24·4%)
-Tertiary School	58 (6·3%)	46 (5%)	104 (5·7%)
**At Boarding School**	60 (6·6%)	61 (6·6%)	121 (6·6%)
**Employment Status**			
-Employed	338 (36·9%)	381 (41·5%)	719 (39·2%)
-In School	177 (19·3%)	170 (18·5%)	347 (18·9%)
-Unemployed	401 (43·8%)	367 (40%)	768 (41·9%)
**Marital Status**			
-Single, never marred	392 (42·8%)	336 (36·6%)	728 (39·7%)
-Married, monogamous	355 (38·8%)	377 (41·1%)	732 (39·9%)
-Married, polygamous	53 (5·8%)	69 (7·5%)	122 (6·7%)
-Widowed	9 (1%)	9 (1%)	18 (1%)
-Divorced	107 (11·7%)	127 (13·8%)	234 (12·8%)
**Number of Children**			
-No Children	384 (41·9%)	381 (41·5%)	765 (41·7%)
-1 Children	299 (32·6%)	282 (30·7%)	581 (31·7%)
-2 Children	156 (17%)	156 (17%)	312 (17%)
-3-5 Children	57 (6·2%)	77 (8·4%)	134 (7·3%)
**Drinks Alcohol**	161 (17·6%)	131 (14·3%)	292 (15·9%)
**Mobile**	251 (27·4%)	296 (32·2%)	547 (29·8%)
**ART regimen at enrollment**			
-TDF/3TC/EFV	699 (77%)	649 (71·6%)	1348 (74·3%)
-TDF/3TC/DTG	95 (10·5%)	122 (13·5%)	217 (12%)
-AZT/3TC/NVP	35 (3·9%)	44 (4·9%)	79 (4·4%)
-TDF/3TC/ATV/r	10 (1·1%)	20 (2·2%)	30 (1·7%)
-AZT/3TC/ATV/r	13 (1·4%)	14 (1·5%)	27 (1·5%)
-ABC/3TC/EFV	13 (1·4%)	11 (1·2%)	24 (1·3%)
-Other	43 (4·7%)	47 (5·2%)	90 (5%)
**Baseline Viral Load <400 copies/mL**	661 (72·6%)	679 (75·1%)	1340 (73·8%)
**Baseline Care Status**			
-Recently Engaged	323 (35·3%)	304 (33·2%)	627 (34·2%)
-Engaged	551 (60·2%)	589 (64·2%)	1140 (62·2%)
-Re-engaging	41 (4·5%)	24 (2·6%)	65 (3·5%)

[1] Restricted to the analytic population of participants who enrolled prior to December 1, 2019 (includes those who later withdrew, transferred or outmigrated before primary endpoint ascertainment). Denominator of all proportions is the number of measured participants; [2] Lived away from home for

more than 1 month of the prior 6 months; [3]Missing for 19 (1·2%) of participants: 8 (0·9%) of intervention and 11 (1·4%) of control. [4] Missing for 19 (1·2%) of participants: 5 (0·6%) of intervention and 14 (1·8%) of control. [5] Missing for 2 (0·1%) of participants: 1 (0·1%) of intervention and 1 (0·1%) of control. [6] Recently engaged: started treatment within 6 months of enrolment or at enrolment. [7] Engaged: started treatment more than 6 months prior to enrolment and had a HIV care visit within 6 months of enrolment. [8] Re-engaging: started treatment more than 6 months prior to enrolment, but did not have a HIV care visit within 6 months of enrolment. TDF: Tenofovir disoproxil fumarate; EFV: efavirenz; AZT: zidovudine; DTG: dolutegravir; NVP: nevirapine; ATV/r: ritonavir booster atazanavir; ABC: abacavir.

Table S5. Clinic-level baseline characteristics, by arm and overall.

Characteristics	Intervention	Control	Total
	N=14	N=14	N=28
# clinics in Kenya	N=7	N=7	N=14
# of youth clients	96 [64,229]	104 [75,218]	104 [68,229]
# enrolled	66 [60,78]	56 [53,81]	62 [54,78]
# in analytic population[1]	56 [47,67]	48 [44,65]	54 [44,68]
Average age	21 [20,21]	21 [20,21]	21 [20,21]
% female	84 [76,87]	79 [74,84]	82 [75,85]
% on TDF/3TC/EFV	77 [71,84]	69 [65,76]	72 [66,82]
% on TDF/3TC/DTG	11 [8,14]	12 [7,17]	11 [7,16]
% with baseline viral suppression[2]	73 [67,81]	77 [72,84]	75 [68,83]
% recently engaged[3]	30 [23,39]	25 [18,34]	28 [20,37]
% engaged[4]	63 [56,72]	72 [63,79]	67 [59,78]
% re-engaging[5]	4 [3,6]	2 [0,3]	3 [2,5]
% engaged with suppression	52 [46,61]	59 [51,63]	55 [49,63]

Metrics are given in clinic-level counts (N=x) or as the median [Q1-Q3] of the clinic-level summaries. [1] The primary analytic population consists of participants who enrolled prior to December 1, 2019 and who did not withdraw from the study, transfer or outmigrate. The remaining clinic-specific summaries are over these participants. [2] Baseline viral suppression: HIV RNA < 400 c/mL. [3] Recently engaged: started treatment within 6 months of enrolment or at enrolment. [4] Engaged: started treatment more than 6 months prior to enrolment and had a HIV care visit within 6 months of enrolment. [5] Re-engaging: started treatment more than 6 months prior to enrolment, but did not have a HIV care visit within 6 months of enrolment. TDF: Tenofovir disoproxil fumarate; EFV: efavirenz; DTG: dolutegravir

Table S6. Arm-specific and intervention effect estimates on virologic suppression when varying the approach to defining the analytic population and analytic approach to missingness

Analysis	Clinic-level endpoint description	Intervention (95%CI)	Control (95%CI)	Effect Size (95% CI)
**Primary**	Proportion with viral suppression, excluding persons who outmigrated[1] or transferred[2]	88% (85-92)	80% (77-84)	1.1 (1.03-1.16); p=0.002
**Secondary**	Proportion with viral suppression, including all participants	82% (78-87)	74% (70-77)	1.12 (1.04-1.2); p=0.002
**Raw**	Proportion with viral suppression, excluding living participants without an endpoint viral load	92% (89-95)	87% (83-90)	1.06 (1.01-1.12); p=0.008
Adjusted	Proportion with viral suppression, adjusting for differences in characteristics between participants with and without an endpoint viral load	92% (89-95)	86% (82-89)	1.08 (1.02-1.13); p=0.004

[1]Outmigrated (moved >3 hours traveling distance from study clinic)
[2]Transferred: formally switched HIV care to new clinic

Table S7: Arm-specific and intervention effect estimates on virologic suppression by baseline demographic group

	Intervention (95%CI)	Control (95%CI)	Effect Size (95%CI)
Women	88% (84-92)	83% (79-86)	1·06 (1-1·13); p=0·026
Men	84% (76-91)	75% (70-81)	1·11 (0·98-1·25); p=0·044
Age 15-19 years	85% (80-91)	76% (69-82)	1·13 (1·01-1·26); p=0·015
Age 20-24 years	88% (85-92)	84% (80-87)	1·05 (0·99-1·11); p=0·040

Figure S1. Alternative access choice among intervention participants, by clinic

a) Off-site appointments

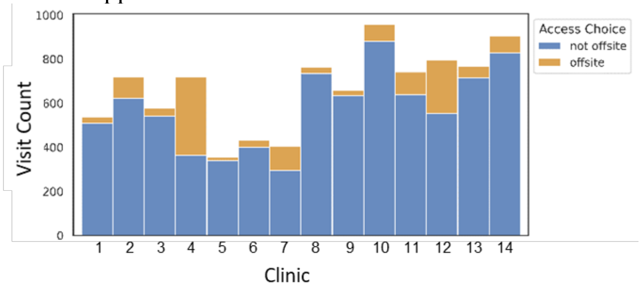

b) Phone appointments

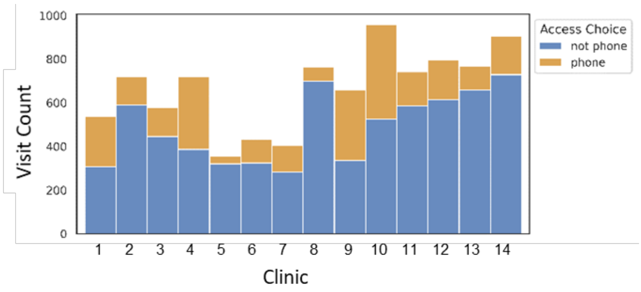

c) Off-hours appointments

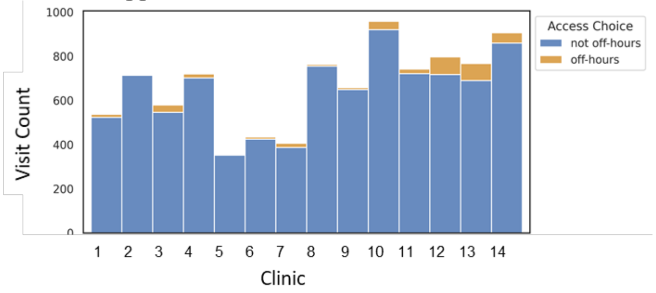

d) Off-site drug delivery

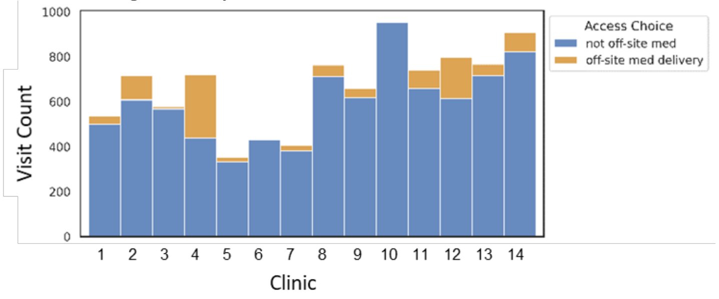

Table S8. Completion and timing of viral load results delivery

Clinic	Results Received	Results Delivered	Delivery Time (mean)	Delivered (%)	Delivered within 3 days (%)
1	357	354	1·18	99%	96%
2	469	463	1·34	99%	96%
3	388	388	1·45	100%	97%
4	469	468	1·36	100%	91%
5	152	151	0·87	99%	97%
6	289	289	0·65	100%	98%
7	252	249	2·81	99%	86%
8	419	415	2·60	99%	85%
9	377	377	0·93	100%	97%
10	542	542	1·59	100%	87%
11	438	438	1·62	100%	94%
12	491	491	2·09	100%	90%
13	607	606	1·19	100%	96%
14	458	458	1·22	100%	94%

Table S9: Annual Cost per Adolescent or Young Adult

	Intervention	Control	Difference
Capital Goods (Gene Xpert)	$ 3·68	$ 3·37	$ 0·31
Facility	$ 10·32	$ 5·15	$ 5·17
Personnel	$ 58·58	$ 33·61	$ 24·97
Recurrent Goods	$ 144·93	$ 101·88	$ 43·05
Services	$ 52·16	$ 117·95	$ (65·79)
Total	$ 269·68	$ 261·97	$ 7·71

Figure S2. Timeline of COVID-19 restrictions affecting COVID-19 clinics during the study period

a) Uganda

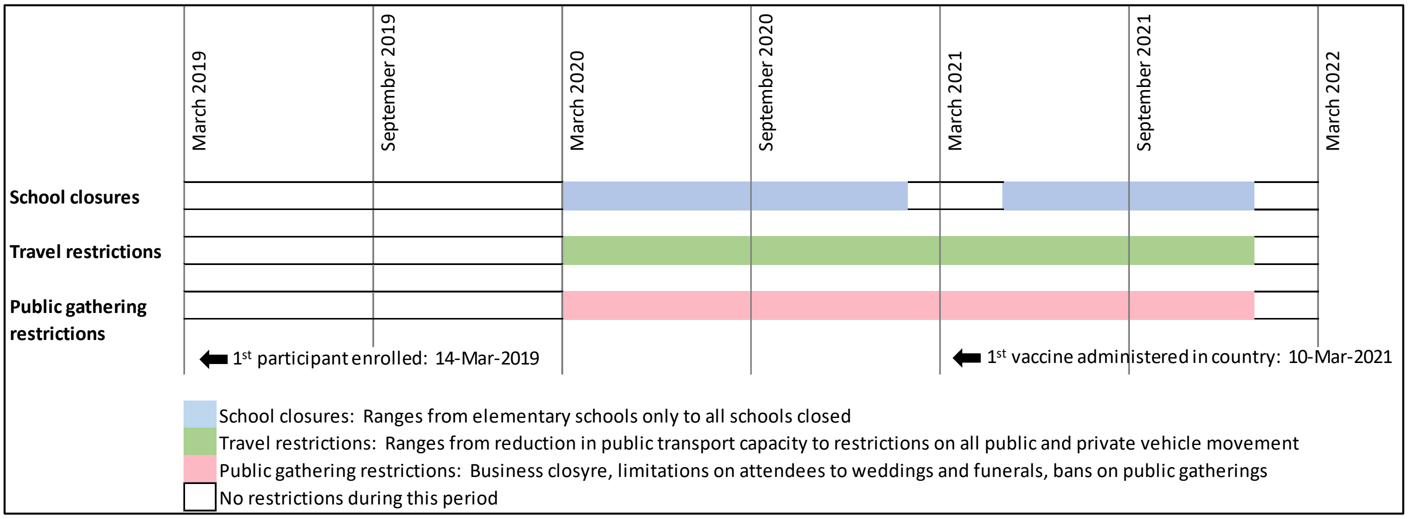

b) Kenya

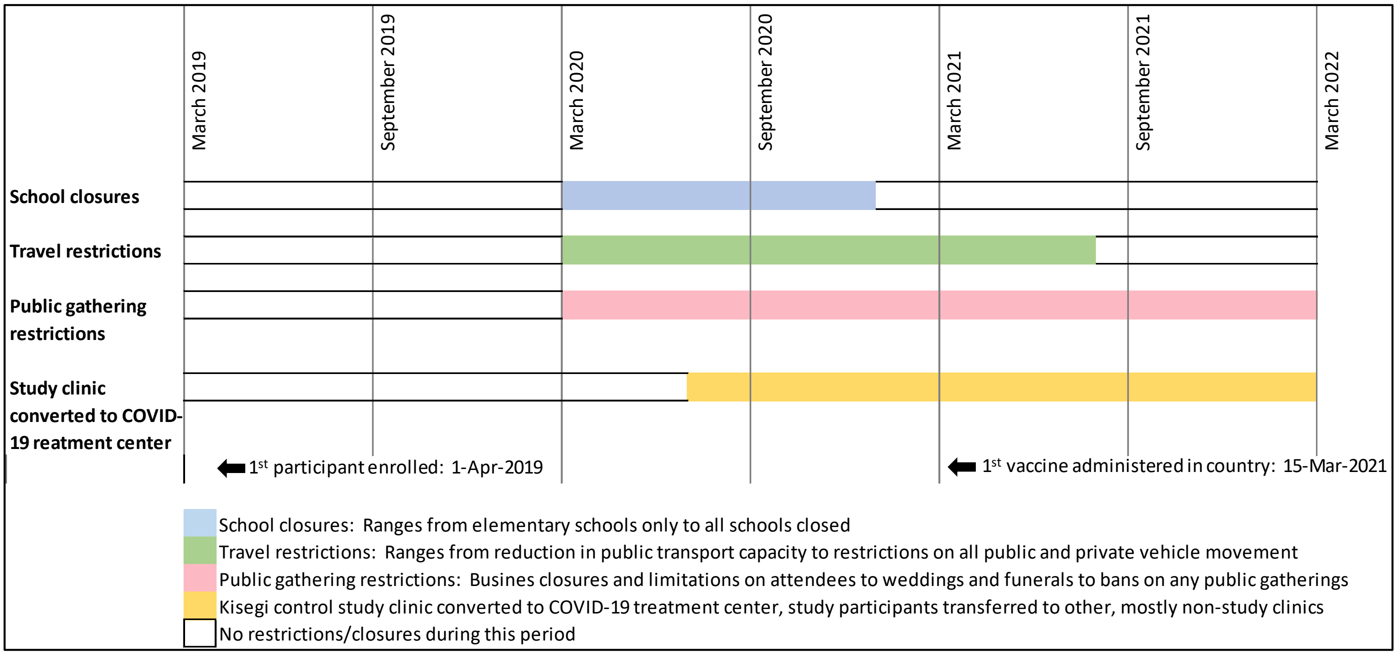

Statistical Analysis Plan
Accessible at the following url: https://arxiv.org/abs/2211.02771

Table S10: CONSORT 2010 checklist of information to include when reporting a cluster randomised trial

Section/Topic	Item No	Standard Checklist item	Extension for cluster designs	Page No *
**Title and abstract**				
	1a	Identification as a randomised trial in the title	Identification as a cluster randomised trial in the title	1
	1b	Structured summary of trial design, methods, results, and conclusions (for specific guidance see CONSORT for abstracts)[i,ii]	See table 2	Abstract (page 2)
**Introduction**				4
**Background and objectives**	2a	Scientific background and explanation of rationale	Rationale for using a cluster design	4
	2b	Specific objectives or hypotheses	Whether objectives pertain to the the cluster level, the individual participant level or both	6
**Methods**				4-6
**Trial design**	3a	Description of trial design (such as parallel, factorial) including allocation ratio	Definition of cluster and description of how the design features apply to the clusters	4
	3b	Important changes to methods after trial commencement (such as eligibility criteria), with reasons		5
**Participants**	4a	Eligibility criteria for participants	Eligibility criteria for clusters	4
	4b	Settings and locations where the data were collected		**4-5**
**Interventions**	5	The interventions for each group with sufficient details to allow replication, including how and when they were actually administered	Whether interventions pertain to the cluster level, the individual participant level or both	5
**Outcomes**	6a	Completely defined pre-specified primary and secondary outcome measures, including how and when they were assessed	Whether outcome measures pertain to the cluster level, the individual participant level or both	5-6
	6b	Any changes to trial outcomes after the trial commenced, with reasons		**5**
**Sample size**	7a	How sample size was determined	Method of calculation, number of clusters(s) (and whether equal or unequal cluster sizes are assumed), cluster size, a coefficient of intracluster correlation (ICC or *k*), and an indication of its uncertainty	6
	7b	When applicable, explanation of any interim analyses and stopping guidelines		**N/A**

**Randomisation:**				4
**Sequence generation**	8a	Method used to generate the random allocation sequence		**4**
	8b	Type of randomisation; details of any restriction (such as blocking and block size)	Details of stratification or matching if used	4
**Allocation concealment mechanism**	9	Mechanism used to implement the random allocation sequence (such as sequentially numbered containers), describing any steps taken to conceal the sequence until interventions were assigned	Specification that allocation was based on clusters rather than individuals and whether allocation concealment (if any) was at the cluster level, the individual participant level or both	4
**Implementation**	10	Who generated the random allocation sequence, who enrolled participants, and who assigned participants to interventions	Replace by 10a, 10b and 10c	
	10a		Who generated the random allocation sequence, who enrolled clusters, and who assigned clusters to interventions	4
	10b		Mechanism by which individual participants were included in clusters for the purposes of the trial (such as complete enumeration, random sampling)	4
	10c		From whom consent was sought (representatives of the cluster, or individual cluster members, or both), and whether consent was sought before or after randomisation	4
**Blinding**	11a	If done, who was blinded after assignment to interventions (for example, participants, care providers, those assessing outcomes) and how		**4**
	11b	If relevant, description of the similarity of interventions		**N/A**
**Statistical methods**	12a	Statistical methods used to compare groups for primary and secondary outcomes	How clustering was taken into account	6
	12b	Methods for additional analyses, such as subgroup analyses and adjusted analyses		**6**
**Results**				6-8
**Participant flow (a diagram is strongly recommended)**	13a	For each group, the numbers of participants who were randomly assigned, received intended treatment, and were analysed for the primary outcome	For each group, the numbers of clusters that were randomly assigned, received intended treatment, and were analysed for the primary outcome	Figure 1

	13b	For each group, losses and exclusions after randomisation, together with reasons	For each group, losses and exclusions for both clusters and individual cluster members	Figure 1
**Recruitment**	14a	Dates defining the periods of recruitment and follow-up		**7**
	14b	Why the trial ended or was stopped		**N/A**
**Baseline data**	15	A table showing baseline demographic and clinical characteristics for each group	Baseline characteristics for the individual and cluster levels as applicable for each group	Table 2 and Table S4
**Numbers analysed**	16	For each group, number of participants (denominator) included in each analysis and whether the analysis was by original assigned groups	For each group, number of clusters included in each analysis	6
**Outcomes and estimation**	17a	For each primary and secondary outcome, results for each group, and the estimated effect size and its precision (such as 95% confidence interval)	Results at the individual or cluster level as applicable and a coefficient of intracluster correlation (ICC or k) for each primary outcome	7; Tables 3-4; Figure 2
	17b	For binary outcomes, presentation of both absolute and relative effect sizes is recommended		
**Ancillary analyses**	18	Results of any other analyses performed, including subgroup analyses and adjusted analyses, distinguishing pre-specified from exploratory		**7**
**Harms**	19	All important harms or unintended effects in each group (for specific guidance see CONSORT for harms[iii])		**N/A**
**Discussion**				9-15
**Limitations**	20	Trial limitations, addressing sources of potential bias, imprecision, and, if relevant, multiplicity of analyses		**11**
**Generalisability**	21	Generalisability (external validity, applicability) of the trial findings	Generalisability to clusters and/or individual participants (as relevant)	11
**Interpretation**	22	Interpretation consistent with results, balancing benefits and harms, and considering other relevant evidence		**3, 9-15**
**Other information**				**Supplementary info**
**Registration**	23	Registration number and name of trial registry		**Abstract; 5**
**Protocol**	24	Where the full trial protocol can be accessed, if available		**N/a**
**Funding**	25	Sources of funding and other support (such as supply of drugs), role of funders		**12**

** Note: page numbers optional depending on journal requirements*

Supplementary Material References

[i] Hopewell S, Clarke M, Moher D, Wager E, Middleton P, Altman DG, et al. CONSORT for reporting randomised trials in journal and conference abstracts. *Lancet* 2008, 371:281-283

[ii] Hopewell S, Clarke M, Moher D, Wager E, Middleton P, Altman DG at al (2008) CONSORT for reporting randomized controlled trials in journal and conference abstracts: explanation and elaboration. *PLoS Med* 5(1): e20

[iii] Ioannidis JP, Evans SJ, Gotzsche PC, O'Neill RT, Altman DG, Schulz K, Moher D. Better reporting of harms in randomized trials: an extension of the CONSORT statement. *Ann Intern Med* 2004; 141(10):781-788.
